# Antioxidant and Free Radical Scavenging Capacity of Seed and Shell Essential Oils Extracted from *Abrus precatorius* (L)

**DOI:** 10.3390/antiox3020278

**Published:** 2014-04-15

**Authors:** Sunday O. Okoh, Olayinka T. Asekun, Oluwole B. Familoni, Anthony J. Afolayan

**Affiliations:** 1 Department of Chemistry, University of Lagos, Yaba, Lagos 234, Nigeria; E-Mails: sunnyokoh2003@yahoo.com (S.O.O.); familonio@unilag.edu.ng (O.B.F.); 2 Department of CFET, Federal Institute of Industrial Research, Lagos 234, Nigeria; 3 Department of Botany, University of Fort Hare, Alice 5700, South Africa; E-Mail: aafolayan@ufh.ac.za

**Keywords:** *Abrus precatorius*, essential oils, DPPH, ABTS, lipid peroxidation, nitric oxide

## Abstract

Essential oils from plants have been proven safe as natural antioxidants, and few are already marketed as digestive enhancers as well as in prevention of several degenerative diseases. This study evaluated the antioxidant capacity of seed and shell essential oils of *Abrus precatorius* (L), a herb used for ethno-medicinal practices in Nigeria. The essential oils were obtained by hydro-distillation. The ability of the oils to act as hydrogen/electrons donor or scavenger of radicals were determined by *in-vitro* antioxidant assays using 2,2-diphenyl-2-picryl-hydrazyl free radical (DPPH^.^) scavenging; 2,2′-azino-bis(3-ethylbenzthiazoline-6-sulfonic acid) (ABTS) radical scavenging; lipid peroxide and nitric oxide radicals scavenging assays. The IC_50_ of the seed and shell oils (2.10 mg/mL and 1.20 mg/mL respectively) showed that antioxidant activity is higher than that for the standard drugs (3.20 mg/mL and 3.40 mg/mL) for the nitric oxide scavenging assay. The lipid peroxidation radical activity of the oils were similar to vitamin C, weak DPPH and ABTS radical scavenging activities were discovered in comparison to vitamin C and rutin. Generally, in the four antioxidant assays, a significant correlation existed between concentrations of the oils and percentage inhibition of free radicals and lipid peroxidation. The composition of *A. precatorius* essential oils reported earlier may account for their antioxidant capacity.

## 1. Introduction

There are increasing evidences that free radicals produced molecular alterations that are associated with various degenerative human diseases such as arteriosclerosis, cancers, Alzheimer’s disease, Parkinson’s disease, diabetes, asthma, arthritis, immune deficiency diseases and aging [1,2,3,4]. Antioxidants are substances that mop up free radicals and prevent them from causing cell damage [5,6]. Plants contain antioxidant compounds that function as free radical scavengers, reducing agents and quenchers of singlet oxygen formation [7]. In the last decade, there have been increasing suggestions and demand for use of essential oils as natural antioxidant than any other plant extracts as potential substitute for the synthetic ones [8,9,10]. Essential oils reduce oxidation within body fluids by inhibiting radicals and increase permeability of microbe’s cell membrane leading to perforation and leakage of cell vital intracellular constituent such as potassium ions (K^+^), adenosine triphosphate (ATP), thus preventing growth of pathogens [5,6]. Essential oil has demonstrated superior properties such as ability to penetrate cell membrane and tissues 100 times faster than water; 10,000 times faster than salt and it can penetrate the blood-brain barrier [9]. Studies have shown that these unique properties are due to volatile, lipophilic nature and chemical structure of essential oil compounds as well as their strong potency as free radical quenching agents [11]. The interest in essential oils and their application in food preservation have been amplified in recent years by an increasingly negative consumer perception of synthetic preservatives. Studies have established that the synthetic antioxidants unlike the natural antioxidants cannot be recycled or re-used by the organism once they have donated their electron to quench free radicals, hence they become harmful metabolic byproducts that increase, rather than decreasing the total load of oxidative stress [12,13]. Essential oils contain a variety of volatile compounds called secondary metabolites, important for plant defense and recent biochemical *in-vivo* studies have characterized some of the compounds as natural antioxidants [14,15,16]. Studies have also shown that some volatile compounds in essential oils possess strong activity against degenerative diseases including very deadly ones such as breast cancer. Limonene and β-caryophyllene isolated from essential oils have been documented to exhibit such activities [15,17,18].

*Abrus precatorius* (L.) belongs to the family Leguiminosae, is commonly called crab eye, and grows in tropical and subtropical climates such as South China, Sri Lanka, Thailand, North America and West Indies [19]. It is prevalent in South West Nigeria. The seeds are considered useful as diuretic, laxative, purgative, emollient, abortifacient and sedative agents. In traditional medicine, it is used to treat itches, sores and wounds. The aqueous and ethanolic extracts of the seed have been reported to contain phenols, tannins, alkaloids, flavonoids and steroids [20]. In our previous reports on the composition of the leaves, seeds and shell of *A. precatorius* plant, monoterpenes, sesquiterpenes, alcohols, phenols and unsaturated fatty acids were documented as the predominant compounds [21]. However, there is dearth of information on the antioxidant capacity, and free radical scavenging activity of the essential oil of the plant. Therefore, this present study was conducted to investigate the antioxidant capacity and free radical scavenging activity of the oils of *A. precatorius* hoping to justify its ethno-medicinal uses and expose more avenues of its exploitation. This work is part of an ongoing research aimed at discovering new essential oil plants with bioactive potentials either as flavors or fragrances that are indigenous to Nigeria.

## 2. Experimental Section

### 2.1. Chemicals Used

Potassium persulfate (PPS), 2,2-dipphenyl-1-picrylhydrazyl (DPPH) and 2,2-azinobis-(3-ethylbenzothiazolin-6-sulfonic acid) diammonium salt (ABTS) were purchased from Sigma-Aldrich (St Louis, MO, USA). Methanol was purchased from Fluka Chemicals (Buchs, Switzerland). All other chemicals used were analytical grade.

### 2.2. Plant Material and Extraction of Essential Oils

The seeds of *A. precatorius* were collected at the plantation of Forestry Research Institute of Nigeria (FRIN), Ibadan, Nigeria. It was authenticated at the Botany Department, University of Lagos by Mr. T. K. Odewo. A voucher of the plant was deposited in the University of Lagos herbarium. After drying, the shells were separated from the seeds and pulverized. Powdered seeds and shells were separately hydrodistilled using the modified Clevenger apparatus. The oil was captured in steam and extracted into *n*-hexane at 100–110 °C for 3 h. The oil extracted in *n*-hexane was carefully dispensed, dried with Na_2_SO_4_ and kept in tinted vials at 4 °C until further analysis. The extraction was carried out several times to get sufficient essential oils for the antioxidant study.

### 2.3. DPPH Assay

The DPPH assay of the essential oil was carried out as previously described [22]. A solution of 0.135 Mm DPPH in methanol was prepared and 1.0 mL of this solution was mixed with 1.0 mL of the essential oil prepared in methanol containing 0.025–0.50 mg/mL of the oil and commercial antioxidants (vitamin C and rutin). The reaction mixture was vortexed thoroughly, left in the dark at 25 °C for 30 min and measured at 517 nm. The ability of the essential oil to scavenge DPPH radical was calculated as % inhibition by the following equation:

% inhibition={(Abs_control_ − Abs_sample_)}/(Abs_control_) × 100 (1)

where Abs control is the absorbance of the DPPH radical + methanol; Abs sample is the absorbance of DPPH radical + essential oil or commercial antioxidant. The inhibitory concentration (IC_50_) of the essential oil needed to inhibit 50% of the DPPH radicals obtained from the standard curve was compared to that of standard/commercial antioxidants (vitamin C and rutin).

### 2.4. ABTS Assay

In the ABTS free radical assay, the method of Witayapan [23] was adopted with minor changes (ABTS stock solution diluted in methanol). The pre-formed radical mono cation of ABTS was generated by oxidation of ABTS solution (7 mM) with 2.45 mM potassium persulfate solution in equal amount. The mixture was allowed to react for 12 h in the dark at 25 °C. 1 mL of the resulting solution was diluted in 60 mL of methanol to obtain an absorbance of 0.706 ± 0.001 at 734 nm. 1 mL of the ABTS radical cation solution was added to the 0.025, 0.05, 0.10, 0.2 and 0.5 mg/mL of essential oil solutions and commercial antioxidants (vitamin C and rutin) prepared in methanol and absorbance measured at 734 nm. The percentage inhibition of ABTS radical by the oils was calculated using the equation described in the DPPH assay.

### 2.5. Lipid Peroxides Radical Scavenging Capacity

The Thiobarbituric acid-reactive species (TBARS) assay described by Badmus [24] was used to measure the lipid peroxidation, using egg-yolk homogenates as lipid-rich media with a minor change (methanol used for dilution of oils instead water). Egg homogenate (0.50 mL, 10% in distilled water, v/v) and the oils 0.025–0.50 mg/mL in methanol were mixed, the volume was made up to 1 mL, by adding methanol. 0.05 mL FeSO_4_ (0.07 M) was added to the mixture and incubated for 30 min, to induce lipid peroxidation. Thereafter, 1.5 mL of 20% acetic acid (pH adjusted to 3.5 with NaOH) and 1.5 mL of 0.8% TBA (w/v) (prepared in 1.1% sodium dodecyl sulfate) and 0.05 mL 20% TCA were added, the test tubes were vortexed and heated in a water bath for 60 min. After cooling, 5.0 mL of 1-butanol was added and centrifuged at 3000 rpm for 10 min. The absorbance of the organic upper layer was measured at 532 nm. For the blank 0.1 mL of methanol was used in place of the oil. The percentage of inhibition of lipid peroxide was calculated using the equation described in the DPPH assay.

### 2.6. Nitric Oxide Radical Scavenging Capacity

The method described by Makhija *et al.* [25] was adopted. Nitric oxides radicals were generated from a sodium nitroprusside solution; Sodium nitroprusside (1 mL of 10 mM) was mixed with 1 mL of oils to give concentrations of 0.025–0.50 mg/mL in phosphate buffer. The mixture was incubated at 25 °C for 150 min. To 1 mL of the incubated solution, 1 mL of Griess’ reagent was added. Absorbance was read at 546 nm. The % inhibition of nitric oxide radical by the oil was calculated using the equation described in the DPPH assay. All measurements were done in triplicates and mean values were calculated.

### 2.7. Statistical Analyses

Data were calculated as means ± SD. Pearson’s correction analysis (SPSS 16.0 for windows, SPSS Inc., Chicago, IL, USA) was used to test for the significance of the relationship between the concentration and percentage inhibition.

## 3. Results and Discussion

The seed and shell essential oils from *A. precatorius* were examined for radical scavenging and antioxidant activities using four different assay methods. The percentage inhibitions of the oils were concentration dependent.

**Figure 1:**
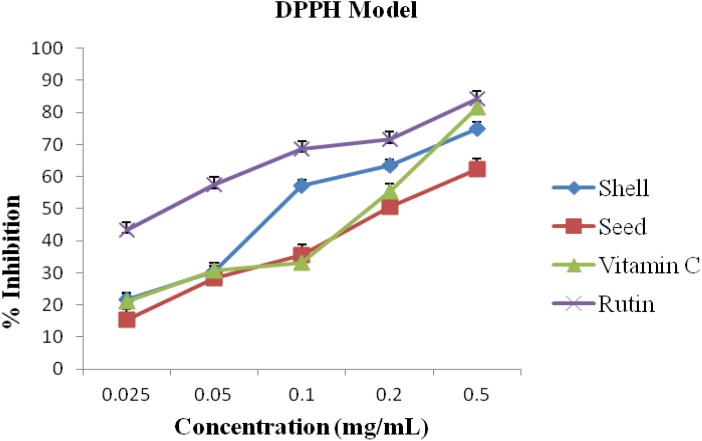
Antioxidant activity of the shell and seed essential oils extracted from *A. precatorius* on 2,2-diphenyl-2-picryl-hydrazyl free radical (DPPH) radicals.

The percentage inhibitions for DPPH assay are given in Figure 1. At all concentrations (0.025–0.5 mg/mL) the shell oil showed stronger DPPH radicals scavenging activity than the seed oil. However, the oils activity at 0.2 mg/mL and 0.5 mg/mL were above average. Overall, rutin demonstrated superior scavenging activity.

**Figure 2:**
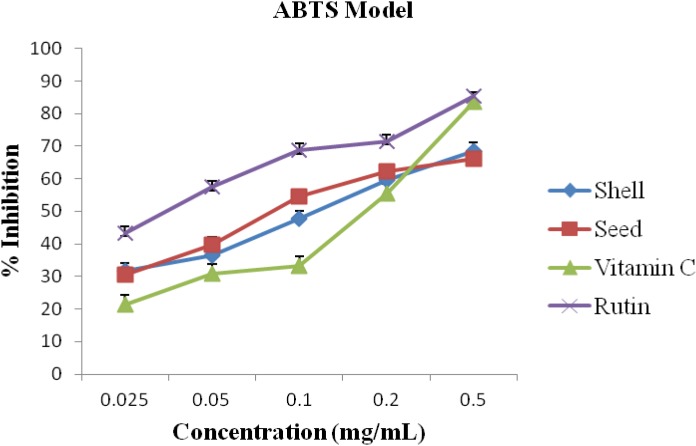
Antioxidant activity of the shell and seed essential oils extracted from *A. precatorius* on 2, 2′-azino-bis (3-ethylbenzthiazoline-6-sulfonic acid) (ABTS) radicals.

The percentage inhibitions pattern of the ABTS radicals was similar to DPPH. However, the seed oil demonstrated stronger inhibitions at concentrations of 0.05 and 0.1 mg/mL than the shell oil (Figure 2). Vitamin C had lower activity than the two oils except at concentration of 0.5 mg/mL.

**Figure 3:**
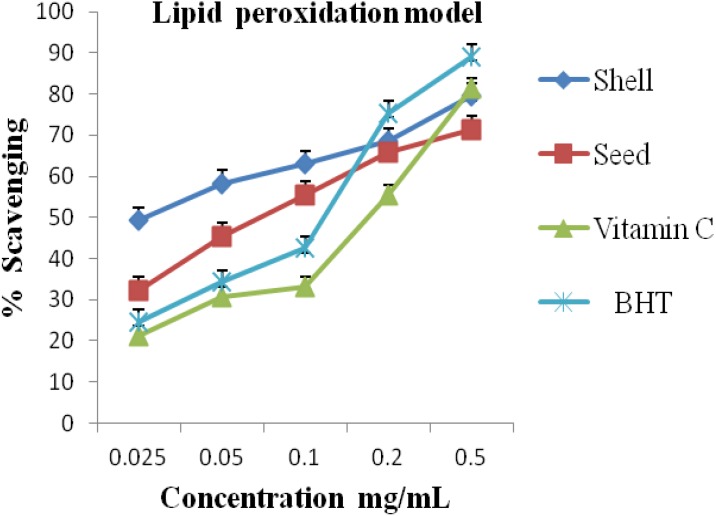
Antioxidant activity of the shell and seed essential oils extracted from *A. precatorius* on lipid peroxide radicals.

The lipid peroxide radicals scavenging activity of the essential oils results at various concentrations is shown in Figure 3. The shell oil displayed a stronger lipid peroxide radicals scavenging activity than the seed oil and the two standard antioxidants at 0.025–0.10 mg/mL. At all concentrations, the shell oil demonstrated superior lipid peroxide radicals scavenging activity than the seed oil. Notable is the significant difference in lipid peroxide radicals scavenging activity between the shell oil and vitamin C.

**Figure 4:**
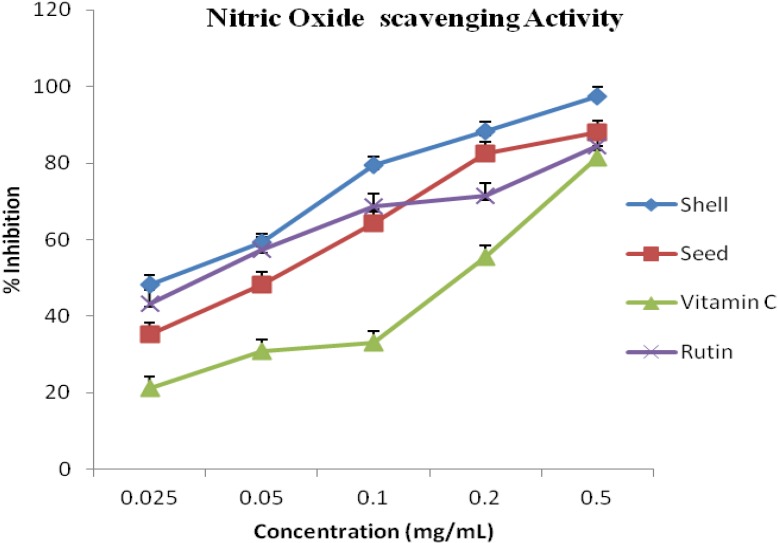
Antioxidant activity of the shell and seed essential oils extracted from *A. precatorius* on nitric oxide radicals.

The radicals scavenging activity of the seed and shell essential oils from *A. precatorius* in nitric oxides rich (NO) medium at various concentrations are presented in Figure 4. The percentages of inhibition of the shell oil were higher than that of the seed oil, this is comparable to the lipid assay result. The activity of the oils was higher than that of vitamin C and comparable to rutin.

It has been established that DPPH test does not discriminate between radical species, but generally ideal for radicals quenching ability [2]. Hence, for quantitative and qualitative antioxidant capacity of the oils, we examined suspected antioxidant activity using four radical quenching assays. In the series of the *in vitro* tests the seed and shell essential oils of *A. precatorius* exhibited significant antioxidant activity by acting as donators of proton or electron in the DPPH, ABTS assays and possessed hydroxyl, lipid peroxide and nitric oxide radical scavenging properties. In all four assays, the shell and seed oils possessed greater activity than vitamin C. These results are presented in Figure 1, Figure 2, Figure 3 and Figure 4, the IC_50_ values for the oils are presented in Table 1.

**Table 1:** Antioxidant capacity of essential oils of *A. precatorius* (mg/mL).

S/N	Activity	*A. precatorius*		Standard/Commercial Antioxidants (Positive Controls)		
		Seed Oil (IC_50_)	Shell Oil (IC_50_)	Vitamin C (IC_50_)	Rutin (IC_50_)	BHT ^b^ (IC_50_)
1	DPPH^•^	5.0 3 ± 0.24	3.03 ± 0.11	1.50 ± 0.01	0.50 ± 0.04	ND
2	ABTS^+•^	2.95 ± 0.31	3.07 ± 0.22	0.10 ± 0.04	0.10 ± 0.01	ND
3	LP^•^	1.92 ± 2.10	1.42 ± 0.40	1.83 ± 0.33	ND	0.83 ± 0.40
4	NO^•^	2.10 ± 0.40	1.20 ± 0.20	1.20 ± 0.20	3.40 ± 0.01	ND

DPPH^•^ = 2,2-diphenylpicrylhydrazyl radicals, ABTS^+•^ = 2,2′-azino-bis diammonium salt radicals, LP^•^ = lipid peroxide radical, NO^•^ = Nitric oxide radical, ^b^ BHT = Butylated hydroxyl toluene, ND = Not determined, the lower IC_50_ (mg/mL) the higher the antioxidant capacity. Values are mean ± SD, *n* = 3.

The seed and shell oils were able to reduce the stable DPPH radical to 50% reduction with IC_50_ of 5.03 ± 0.24 and 3.03 ± 0.11 mg/mL respectively. The antioxidant capacity of the two oils in the DPPH radicals assay were lower than those of the standard antioxidants (vitamin C and rutin). The seed oil was more effective in scavenging ABTS radicals than the DPPH radicals with IC_50_ values 2.95 ± 0.31 and 5.03 ± 0.24 mg/mL respectively, this might be due to the fact that the complexity of essential oils, polarity and chemical properties, could lead to varying bioactivity results depending on the method adopted [26]. The shell oil activity in both assays was similar (3.03 ± 0.11 and 3.07 ± 0.22 mg/mL). The shell oil was able to inhibits the lipid peroxides radicals more than the seed oil and vitamin C with IC_50_ value of 1.42 ± 0.40 mg/mL. However, BHT demonstrated significantly higher antioxidant capacity (0.83 ± 0.4 mg/mL). In the nitric oxide radical test, the oils and standards effectively reduced the generated NO radicals. They exhibited strong NO radical scavenging capacity with valuable IC_50_ of 2.10 ± 0.40, 1.20 ± 0.20 and 3.4 ± 0.01, 3.2 ± 0.02 mg /mL respectively. Interestingly, the shell and seed oils displayed superior capacity than vitamin C and rutin. Factors like stereo-selectivity of the radicals or the solubility of the oil in the different testing systems have also been reported to affect the capacity of essential oils in quenching different radicals [26]. Wang *et al.* [12] reported that some compounds, which have ABTS radicals scavenging activity, do not show DPPH activity. Hence, the ability of the seed and shell essential oils of *A. precatorius* to scavenge different free radicals in different systems is noteworthy. This indicates that the oils may be useful for preventing radical related pathological damage, especially breakdown of biomolecules and DNA by LP and NO radicals that may lead to arteriosclerosis, carcinogenesis and inflammation [27]. Several studies have shown that chronic expression of NO radicals is associated with inflammation conditions including juvenile diabetes, multiple sclerosis, arthritis, ulcerative colitis [28,29]. The GC/MS quantitative and qualitative investigation of the leaf, seed and shell essential oils of *A. precatorius* in our previous report [21], revealed the presence of terpenes, phenol, alcohols, terpenoids and unsaturated fatty acids as prominent compounds. The major monoterpenes compounds in shell oil were limonene (19.08%), ocimene (8.94%) and myrcene (8.60%), while zingiberene (6.02%) and β–bisabolene (3.92%) were the prominent sesquiterpenes. Other important compounds identified in the shell oil are, camphene (6.75%), α–pinene (4.18%), curcumene (3.10%), α–terpineol (2.34%) and methyl eugenol (0.26%). In seed oil the dominant monoterpenes are sabinene (10.93%) and camphene (6.45%), while were zingiberene (10.75%), farnesene (5.30%), sesquiphelladrene (4.47%) and curcumene (4.41%) were the prominent sesquiterpenes. Two unsaturated fatty (9,12-octadecedienoic 10.92% and 10-octadecenoic 3.16%) acids were also compounds identified in the seed oil. These compounds could be responsible for the substantial antioxidant capacity demonstrated by the essential oils, suggesting possible additive or synergist effects of the constituents [30]. The findings in this study are in agreement with previous essential oil researchers, that some essential oils are strong natural antioxidants [31,32].

## 4. Conclusions

This study shows that besides the traditional uses of the plant extract, the essential oils extracted from *A. precatorius* seeds and shells have good antioxidant potential, and could probably replace synthetic antioxidants in further studies.

## References

[1] Saikat S., Chakraborty R., Sridhar C.Y., Reddy S.R., Biplab D. (2010). Free radicals, antioxidants, diseases and phytomedicine: Current status and future prospect. Int. J. Pharm. Sci. Rev. Res..

[2] Sachdev S., Davies K. (2008). Production, detection, and adaptive responses to free radicals in exercise. Free Radic. Biol. Med..

[3] Mahmood R.M., Soheila M., Saeid A. (2008). Radical scavenging and reducing power of *Salvia mirzayanii* subfractions. Molecules.

[4] Mimica-Dukic N., Dušan B., Slavenko M., Dragana V.G., Branka O.D. (2010). Essential oil of *Myrtus communis* L. as a potential antioxidant and antimutagenic agents. Molecules.

[5] Paul S. (2011). *Trachyspermum ammi* (L.). Fruit essential oil influencing on membrane permeability and surface characteristics in inhibiting food-borne pathogens. Food Control.

[6] Morten H., Mygind T., Rikke L. (2012). Essential oils in food preservation: Mode of action, synergies, and interactions with food matrix components. Front. Microbiol..

[7] Millogo-Kone M., Lompo F., Nacoulma O. (2009). Evaluation of flavonoids, total phenolic contents of *P. biglobosa* and free radical scavenging and antimicrobial activities. Res. J. Med. Sci..

[8] Brenes A., Roura E. (2010). Essential oils in poultry nutrition: Main effects and modes of action. Anim. Feed Sci. Technol..

[9] Nweze E.I., Okafor J.I. (2010). Activities of a wide range of medicinal plants and essential oil against *Scedospaorium* isolates. Am. Eurasian J. Sci. Res..

[10] Tuttolomondo T., Virga G., Curcuruto G., la Bella S., Leto C., Licata M., Napoli E., Pasquale A., Saija A., Siracusa L. (2013). Biomolecular characterization of wild sicilian oregano: Phytochemical screening of essential oils and extracts, and evaluation of their antioxidant activities. Chem. Biodivers..

[11] Mansoub N., Myandoab P. (2012). Research opinion in animal. Vet. Sci..

[12] Wang W., Wu N., Zu G., Fu Y. (2008). Antioxidant activity of *Rosmarinus officinalis* (L), essential oil compared to its main components. Food Chem..

[13] Miller E.R., Pastor-Barriuso R., Dalal D., Riemersma R.A., Appel L.J., Guallar E. (2005). Vitamin E supplementation may increase all-cause mortality. Ann. Intern. Med..

[14] Jesudoss V.A., Jayaraman J., Madhavan S. (2012). d-limonene alleviates insulin resistance and oxidative stress in rat. Eur. J. Nutr..

[15] Horvathova E., Katarina K., Srancikova A., Hunakova L., Galova E., Sevcovicova A., Slamenova D. (2012). Borneol administration protects primary rat hepatocytes against exogenous oxidative DNA damage. Mutagenesis.

[16] Bidinotto L.T., Costa C.A., Salvadori D.M., Costa M., Rodrigues M.A., Barbisan L.F. (2011). Effects lemongrass oil on DNA damage and carcinogenesis in female mice. J. Appl. Toxicol..

[17] Kaurinovic B., Vlaisavljevic S., Popovic M., Vastag D., Djurendic-Brenesel M. (2010). Antioxidant properties of *Marrubium peregrinum* essential oil. Molecules.

[18] Avlessi F., Alitonou G.A., Sohounhloue D.K., Bessiere J.M., Menut C. (2005). Chemical and Biological evaluation of leaf essential oil of *Commiphora africana* from Benin. J. Essent. Oil Res..

[19] John D.B., Benjamin P.J., Rathna K., Herin D.S. (2012). *Abrus precatorius* L.: A medicinal plant with potential as antibacterial agent. J. Pharm. Res..

[20] Pal R.S., Ariharasivakumar G., Girhepunje K., Upadhyay A. (2009). *In vitro* antioxidant activity of phenolic and flavonoids compounds extracted from seeds of *Abrus precatorius*. Int. J. Pharm. Pharm. Sci..

[21] Okoh S.O., Asekun O.T., Familoni O.B. (2008). The essential oils and chemical composition of leaf, seed and shell of *Abrus precatorius* from Nigeria. Proceedings of the 4th Annual Research Conference and Fair.

[22] Okoh S.O., Asekun O.T., Familoni O.B., Afolayan A.J. (2011). Composition and antioxidant activities of leaf and root volatile oils of *Morida lucida*. J. Nat. Prod. Commun..

[23] Witayapan N., Sombat C., Siriporn O. (2007). Antioxidant and antimicrobial activities of *Hyptis suaveolens* essential oil. Sci. Pharm..

[24] Badmus J.A., Odunola O.A., Obuotor E. (2010). Phytochemical and *in vitro* antioxidant potentials of *Holarrhena floribunda* leaf. Afr. J. Biotechnol..

[25] Makhija I.K., Aswatha Ram H.N., Shreedhara C.S., Vijay Kumar S., Devkar R. (2011). *In vitro* antioxidant studies of *Sitopaladi Churna*, a polyherbal Ayurvedic formulation. Free Radic. Antioxid..

[26] Guerrini A., Sacchetti G., Rossi D., Paganetto G., Muzzoli M., Andreotti E.  (2009). Bioactivities of *Piper aduncum* L. and *Piper obliquum* Ruiz & Pavon (Piperaceae) essential oils from Eastern Ecuador. Environ. Toxicol. Pharmacol..

[27] Valko M., Leibfritz D., Moncol J., Mazur M., Telser J. (2007). Free radicals and antioxidants in normal physiological functions and human disease. Int. J. Biochem. Cell Biol..

[28] Sathyavathi A., Suchetha N., Vijay R., Ullal D., Praveen A. (2012). Status of Phosphodiesterase, Nirtic oxide and Arginase levels in hypo and hyperthyroidism. Int. J. Res. Pharm. Biomed. Sci..

[29] Fini M.A., Johnson R.J., Stenmark K.R., Wright R.M. (2013). Hypertension, nitrate-nitrite, and xanthine oxidoreductase catalyzed nitric oxide generation: Pros and Cons. Hypertension.

[30] Luh S., Wong S., El-shimi E. (2007). Effect of processing on some chemical constituents of Pistachio nuts. J. Food Qual..

[31] Foti M.C., Ingold K.U. (2009). Unexpected superoxide dismutase antioxidant activity of Ferric chloride in acetonitrile. J. Org. Chem..

[32] Peana A., Marzocco S., Popolo A. (2006). Linalool inhibits *in vitro* NO formation: Probable involvement in the antinociceptive activity of this monoterpene compound. Life Sci..

